# Antifungal and antibiofilm effects of probiotic *Lactobacillus salivarius*, zinc nanoparticles, and zinc nanocomposites against *Candida albicans* from Nile tilapia (*Oreochromis niloticus*), water and humans

**DOI:** 10.3389/fcimb.2024.1358270

**Published:** 2024-06-04

**Authors:** Nashwa El-Gazzar, Rasha M. M. Abou Elez, Amira S. A. Attia, Abdel-Wahab A. Abdel-Warith, Manal M. Darwish, Elsayed M. Younis, Rehab A. Eltahlawi, Kawthar Ibraheem Mohamed, Simon J. Davies, Ibrahim Elsohaby

**Affiliations:** ^1^ Department of Botany and Microbiology, Faculty of Science, Zagazig University, Zagazig, Egypt; ^2^ Department of Zoonoses, Faculty of Veterinary Medicine, Zagazig University, Zagazig, Egypt; ^3^ Department of Veterinary Public Health, Faculty of Veterinary Medicine, Zagazig University, Zagazig, Egypt; ^4^ Department of Zoology, College of Science, King Saud University, Riyadh, Saudi Arabia; ^5^ Medical Microbiology Department, Faculty of Medicine, Ain Shams University, Cairo, Egypt; ^6^ Microbiology and Immunology Department, Faculty of Pharmacy, October University for Modern Sciences and Arts, Giza, Egypt; ^7^ Microbiology and Immunology Department, Faculty of Medicine, Zagazig University, Zagazig, Egypt; ^8^ Aquaculture Nutrition Research Unit ANRU, Carna Research Station, Ryan Institute, College of Science and Engineering, University of Galway, Galway, Ireland; ^9^ Department of Infectious Diseases and Public Health, Jockey Club College of Veterinary Medicine and Life Sciences, City University of Hong Kong, Hong Kong, Hong Kong SAR, China; ^10^ Centre for Applied One Health Research and Policy Advice (OHRP), City University of Hong Kong, Hong Kong, Hong Kong SAR, China; ^11^ Department of Animal Medicine, Faculty of Veterinary Medicine, Zagazig University, Zagazig, Egypt

**Keywords:** *Candida albicans*, virulence genes, zinc nanoparticles, probiotic, nanocomposite, antibiofilm activity

## Abstract

**Introduction:**

*Candida albicans* (*C. albicans*) can form biofilms; a critical virulence factor that provides effective protection from commercial antifungals and contributes to public health issues. The development of new antifungal therapies, particularly those targeting biofilms, is imperative. Thus, this study was conducted to investigate the antifungal and antibiofilm effects of *Lactobacillus salivarius* (*L. salivarius*), zinc nanoparticles (ZnNPs) and nanocomposites (ZnNCs) on *C. albicans* isolates from Nile tilapia, fish wash water and human fish sellers in Sharkia Governorate, Egypt.

**Methods:**

A cross-sectional study collected 300 samples from tilapia, fish wash water, and fish sellers (100 each). Probiotic *L. salivarius* was immobilized with ZnNPs to synthesize ZnNCs. The study assessed the antifungal and antibiofilm activities of ZnNPs, *L. salivarius*, and ZnNCs compared to amphotericin (AMB).

**Results:**

*Candida* spp. were detected in 38 samples, which included *C. albicans* (42.1%), *C. glabrata* (26.3%), *C. krusei* (21.1%), and *C. parapsilosis* (10.5%). A total of 62.5% of the isolates were resistant to at least one antifungal agent, with the highest resistance to nystatin (62.5%). However, 75% of the isolates were highly susceptible to AMB. All *C. albicans* isolates exhibited biofilm-forming capabilities, with 4 (25%) isolates showing strong biofilm formation. At least one virulence-associated gene (*RAS1*, *HWP1*, *ALS3*, or *SAP4*) was identified among the *C. albicans* isolates. Probiotics *L. salivarius*, ZnNPs, and ZnNCs displayed antibiofilm and antifungal effects against *C. albicans*, with ZnNCs showing significantly higher inhibitory activity. ZnNCs, with a minimum inhibitory concentration (MIC) of 10 µg/mL, completely reduced *C. albicans* biofilm gene expression. Additionally, scanning electron microscopy images of *C. albicans* biofilms treated with ZnNCs revealed asymmetric, wrinkled surfaces, cell deformations, and reduced cell numbers.

**Conclusion:**

This study identified virulent, resistant *C. albicans* isolates with strong biofilm-forming abilities in tilapia, water, and humans, that pose significant risks to public health and food safety.

## 1 Introduction

*Candida albicans* (*C. albicans*) is a dimorphic fungus that causes a significant portion (50–90%) of candidiasis cases in humans (Romaní et al., 2006; Kadosh and Lopez-Ribot, 2013; Sardi et al., 2013; Salvatori et al., 2016; Tsui et al., 2016). It colonizes the mucosal surfaces and skin of healthy humans, animals, and fish (Cannon et al., 1995; Koh et al., 2008; Gow et al., 2011). Clinical manifestations of candidiasis range from localized to invasive and systemic disease, depending on individual’s immune status (Papon et al., 2013). *C. albicans* can cause life-threatening infections, especially infections caused by drug-resistant strains, in immunocompromised patients (Pfaller et al., 2010), resulting in significant morbidity, high mortality, and increased healthcare costs (Sherry et al., 2017). Other *Candida* spp., such as *C. tropicalis*, *C. glabrata* and *C. parapsilosis*, cause 30 to 54% of candidiasis cases (Silva et al., 2009; Donadu et al., 2021). *Candida* spp. can also cause acute diseases when contaminating potable water and are increasingly recognized as an emerging cause of chronic water quality problems (Montagna et al., 2012; Montagna et al., 2013; Mhlongo et al., 2019) as they are natural inhabitants of water and can thrive in low circulation areas of water systems (Caggiano et al., 2020).

Several factors, including the host’s immune status, virulence-associated genes, and biofilm formation ability, contribute to the transition of *Candida* spp. from commensal to pathogenic form (Spellberg, 2008; Galocha et al., 2019). The pathogenicity of *Candida* spp. is primarily linked to specific virulence genes, such as agglutinin-like sequence genes (*ALS3*), secretory aspartate protease (*SAP4*), hyphal wall protein 1 (*HWP1*), and a hyphal-regulated gene (*HYR1*), which facilitate the invasion of host tissues (Dwivedi et al., 2011; Mayer et al., 2013; de Oliveira et al., 2017; Bu et al., 2022). These genes are essential for mycelial formation, adhesion, and the development of *C. albicans* biofilm (Schild et al., 2011; de Oliveira et al., 2017; Bu et al., 2022).

*C. albicans* biofilm consist of diverse three-dimensional combinations of hyphae and yeast embedded extracellularly by large numbers of matrix units (Douglas, 2002; Lohse et al., 2018). *C. albicans* can form biofilms on the surfaces of implantable medical instruments and is associated with device-related nosocomial infections. These *Candida* biofilms exhibit resistance levels 30 to 2000 times higher than those of planktonic cells to various antifungal agents, such as fluconazole, ketoconazole, itraconazole and amphotericin (Seneviratne et al., 2008), as well as reduced susceptibility to host immune reactions (da Costa et al., 2009; Berman and Krysan, 2020). The widespread and prolonged use of conventional antimicrobial agents has led to the emergence of fungal drug fights (Abdel-Shafi et al., 2020; Enan et al., 2020; Judan Cruz et al., 2021). Researchers are now exploring innovative strategies to combat multidrug-resistant microbial strains, including the use of nanomaterials (El-Gazzar and Ismail, 2020; Ismail et al., 2020a; Sitohy et al., 2021) and probiotics (Osman and El-Gazzar, 2021), as the development of new antibiotics is challenging.

Nanomaterials composed of various substances, including lipids, metals, and natural or synthetic polymers, serve as efficient drug carriers (El-Gazzar and Enan, 2020). Their rapid and effective biological absorption compared to larger macromolecules makes them excellent materials for delivery systems (El-Bahr et al., 2021; El-Gazzar et al., 2021). In recent years, zinc nanoparticles (ZnNPs) have garnered significant attention as one of the most extensively studied nanostructures produced through nanotechnology, known for their antimicrobial properties against bacteria and fungi. ZnNPs disrupt fungal membranes, leading to cell death by compromising cell structure and function (Hosseini et al., 2019). On the other hand, probiotics, which are diverse antimicrobial substances produced by lactic acid bacteria (LAB) strains, can inhibit *C. albicans* proliferation, adhesion, and biofilm formation (Matsubara et al., 2016; Osman and El-Gazzar, 2021).

Several studies have shown that *Lactobacillus rhamnosus*, *Lactobacillus reuteri*, and *Lactobacillus salivarius* (*L. salivarius*) possess potent antifungal activity against *C. albicans* infection (De Gregorio et al., 2019; Vazquez-Munoz and Dongari-Bagtzoglou, 2021; El-Ashmony et al., 2023). Supernatants of *L. salivarius* demonstrate antifungal effects against *Candida* spp., with lactic acid accumulation in the medium (Zangl et al., 2019). Additionally, organic acids produced by *Lactobacillus* spp. enhance the efficacy of antifungal agents by increasing fungal plasma membrane permeability, facilitating azole uptake. However, lactic acid, low pH, and other secreted metabolites are environmental signals sensed by *C. albicans*, triggering changes in gene expression and the transition to hyphal growth. Another potential mechanism for the probiotic effect could involve competition for available niches and reduced adhesion (Krzyściak et al., 2017). Therefore, the present study aimed to (i) detect the occurrence of *Candida* spp. in tilapia fish, fish wash water, and fish sellers in Sharkia Governorate, Egypt, (ii) identify the virotypes and genotypes of the isolated *C. albicans*, (iii) assess the susceptibility to antifungal drugs and biofilm forming abilities of the identified *C. albicans*, and (iv) investigate the antifungal and antibiofilm effects of *L. salivarius* and ZnNPs and nanocomposites (ZnNCs) on isolates of *C. albicans*.

## 2 Materials and methods

### 2.1 Study design and sampling

A cross-sectional study collected 300 samples from Nile tilapia (*Oreochromis niloticus*), fish wash water, and human fish sellers (100 from each) at various retail fish markets in Sharkia Governorate, Egypt, between April 2022 and January 2023. Sterile cotton swabs were used to collect samples from fish and sellers. The swabs gently rubbed the fish surface and the human seller’s hand before being immersed in tubes containing buffered peptone water (BPW). Additionally, 100 mL of fish wash water was collected in sterile screw-capped glass bottles. The specimens were labeled with the sample identifier, sample type, and date of sampling, and then transported to the laboratory for later testing.

### 2.2 Isolation and biochemical identification

All samples were cultured on Sabouraud Dextrose Agar (SDA; CONDA, Spain) slopes supplemented with 0.05 µg/mL of chloramphenicol and incubated for 24-72 h at 37°C (Amin et al., 2014). Any visible growth on the SDA slopes was further identified through microscopic examination, Gram staining, germ tube testing, and urea hydrolysis testing (Deorukhkar and Roushani, 2018). Pasty and creamy colonies displaying Gram-positive staining and a negative urea hydrolysis test were purified and inoculated into CHROM agar for 48 h at 37°C (Nadeem et al., 2010). Colonies exhibiting a light green color were presumptively identified as *C. albicans* and preserved on SDA slopes at 4°C for further characterization.

### 2.3 Molecular characterization of *C. albicans*

Suspected *C. albicans* colonies were confirmed using PCR targeting the *ITS1* region of the *C. albicans* genome. DNA was isolated with a QIAamp DNA Mini Kit (QIAGEN GmbH, Hilden, Germany) following the manufacturer’s guidelines. The PCR master mix (25 μL) included 12.5 μL of 2X Dream Taq Green master mix kit, 5.5 μL of PCR grade water, 1 μL of both forward and reverse primers (20 pmoL, each), and 5 μL of DNA template. The primers used were obtained from Metabion (Germany) and the PCR conditions are described in **Supplementary Table S1**. Molecularly confirmed *C. albicans* isolates were subjected to virotyping by targeting the *ALS3* gene (Tsang et al., 2012), *HWP1* gene (Inci et al., 2013), *RAS1* gene (Tsang et al., 2012) and *SAP4* gene (Sikora et al., 2011). Positive controls (*C. albicans* ATCC 90028) were included in the PCR assay with the tested strains.

Genotyping of *C. albicans* was performed using RAPD-PCR fingerprinting with the primer OPA-18 following a previously described method (Bautista-Muñoz et al., 2003). The RAPD-PCR fingerprinting information was presented as a binary code based on the absence or presence of every band. The discriminatory power of RAPD-PCR was assessed using Simpson’s index of diversity (D), as previously described (Hunter and Gaston, 1988).

### 2.4 Antifungal susceptibility test

The antifungal susceptibility of *C. albicans* strains was assessed through Kirby-Bauer Disc Diffusion procedure with Mueller Hinton agar. Inhibition zones were determined and interpreted using the Clinical and Laboratory Standards Institute instructions (CLSI, 2008). Eight antifungal agents, including amphotericin (AMB; 20 μg), nystatin (NYS; 100 I.U.); fluconazole (FLZ; 25 μg), itraconazole (ITR; 10 μg), flucytosine (FUS; 1 μg), caspofungin (CAS; 5 μg), micafungin (MIC; 1 μg) and terbinafine (TER; 30 μg), were tested. *C. albicans* ATCC 90028 was used as a quality control. The multiple antifungal resistance (MAR) index was calculated (Cavalcanti Filho et al., 2017). Multidrug resistance (MDR) was also determined (Sanglard, 2016).

### 2.5 Biofilm formation and quantification

Biofilm production of the *C. albicans* strains was assessed using the microtiter plate method (Pierce et al., 2008; Dhanasekaran et al., 2014). A loopful of colonies from fresh agar plates was cultured in SDB media (Oxoid Ltd., Cambridge, UK), overnight at 30°C at 150 rpm in an orbital shaker (Lab-line Incubator Shaker; Elliott Bay Laboratory Services Inc., Seattle, WA, USA). The yeast cells were harvested by centrifugation at 3,000 rpm for 10 min, washed twice in sterile phosphate-buffered saline (PBS, Sigma-Aldrich, St. Louis, MO., USA), resuspended in RPMI 1640, counted using a hemocytometer, and concentration-adjusted to 1 × 10^6^ cells/mL. A 100 μL aliquot of cell suspension (*C. albicans* + SDB) was inoculated into individual well of the 96 well-flat bottom microtiter plates in triplicate. Wells of every plate are containing SDB that were recognized as negative controls. The plates were incubated for a day at 37°C, before being washed three times with sterile 200 µL PBS. The plates were inverted, and PBS was removed. The biofilms were subsequently fixed with 150 μL of 95% ethanol for 20 min. Finally, the biofilm was stained with 0.1% (w/v) crystal violet and left at room temperature for 15 min, then washed twice with PBS and left for 1 h to dry.

The optical density (OD) of stained adherent *C. albicans* biofilm was measured at wavelength of 590 nm by an ELISA reader subsequent to the negative control (OD_NC_) at zero. The means and standard deviations of OD values were recorded for all *C. albicans* isolates, positive (*C. albicans*, ATCC 90028) and negative controls. The isolates were then considered to be non-biofilm formers (OD_590_ ≤ OD_NC_), weak (OD_NC_< OD_590_ ≤ 2 x OD_NC_), moderate (2 × OD_NC_< OD_590_ ≤ 4 × OD_NC_), or strong (4 × OD_NC_< OD_590_) biofilm formers.

### 2.6 DNA sequencing and phylogenetic analysis

The DNA products amplified from two MDR and strong biofilm-forming *C. albicans* strains were isolated with a QIAquick PCR Kit (QIAGEN, Valencia, CA, USA), following the manufacturer’s guidelines. *ITS* gene sequencing was carried out with primers (**Supplementary Table S1**) and an automated sequencer, as previously described (Tarini et al., 2010).

The obtained sequences were resolved with the Basic Local Alignment Search Tool (BLAST^®^ analysis) of Informative Biotechnology website on the National Center. Gene sequences of the *C. albicans* isolates were submitted to the GenBank sequence database under accession numbers OQ150021 and OQ150022. Initial BLAST^®^ analysis was performed to establish sequence identity with GenBank accessions (Altschul et al., 1990). These sequences were then aligned with others available in the GenBank sequence database. A phylogenetic tree was constructed using MEGA 6 (Tamura et al., 2013).

### 2.7 Effects of probiotic *L. salivarius*, ZnNPs and ZnNCs on *C. albicans*

#### 2.7.1 Biosynthesis of ZnNPs and ZnNCs

ZnNPs biosynthesis followed the protocol previously described by El-Gazzar and Ismail (2020). It involved using a 1 mM salt of ZnO_2_ metal (Nanotech for Photo-Electronic Co., Dreamland, 6-October, Egypt) with enzyme extract from *Aspergillus fumigatus*. The mixture was then incubated at 28°C until ZnNPs biosynthesis was complete.

The probiotic strain *L. salivarius* DSM 20555 from the Belgian Co-ordinated Collection of Microorganisms (BCCM) was cultivated in Mane Rogosae Sharp (MRS) broth (Oxoid, Roskilde, Denmark). Probiotic solution (1 mL) was applied to MRS broth (9 mL) and incubated for 24 h at 37°C. Probiotic cultures were centrifuged after incubation for 10 min at 4000 rpm, and the supernatant was collected and purify-sterilized by a 0.2 mm membrane syringe filter.

The synthesis of ZnNCs was carried out through a sono-chemical method, in which 1 g of ZnNPs mixed in 200 mL of deionized distilled water was added to a 250 mL beaker with 1 mL of probiotic *L. salivarius* and subjected to sonication under conditions of 0.5 cycle and 50% amplitude for 2 h until a homogeneous mixture was obtained (Ismail et al., 2020b).

#### 2.7.2 Characterization of ZnNPs and ZnNCs

ZnNPs and ZnNCs were characterized using dynamic light scattering (DLS) to determine the particle diameter (El-Gazzar and Ismail, 2020). Additionally, transmission electron microscopy (TEM) was performed on both ZnNPs and ZnNCs. In a solution of double-deionized water, ZnNPs and ZnNCs were added and then sonicated for 50 min at a frequency of 50 kHz with a cycle length of 0.65 and amplitude of 85% (UP400S, Hielscher, Germany). The resulting slurry was aliquoted (5 µL) and applied to a copper grid with carbon coating. TEM analysis was conducted using a TEM-2100 (JEOL, Tokyo 196-8558, Japan) (Sitohy et al., 2021).

X-ray diffraction (XRD) test was performed using a Bruker D8 Discovers Set (Billerica, MA, USA) to determine the colloidal nature and evaluate the homogeneity and reunify of the synthesis methods for ZnNPs, probiotic *L. salivarius*, and ZnNCs. The probiotic *L. salivarius* and ZnNCs were subjected to assessment via Energy Dispersive-X-ray Spectroscopy (EDS) (Sitohy et al., 2021).

#### 2.7.3 Antibiofilm effect

A suspension (100 μL) of *C. albicans* (1 × 10^6^ cells/mL) was plated in 96-well microtiter dishes as described earlier and incubated with agitation. The effects of ZnNPs, probiotic *L. salivarius*, ZnNCs, and antifungal (AMB) on hyphal development and the early phases of biofilm consistence (preventative) were assessed. Free floating cells were removed 1.5 h after adhesion by thoroughly washing each well with 200 μL of PBS. Subsequently, after another 24 h at 37°C with agitation, adhesive cells were exposed to 200 μL of various doses of the test drugs that had been diluted on a separate microtiter dish.

The produced biofilm biomass was quantified for ZnNPs, probiotic *L. salivarius*, ZnNCs, and AMB in comparison to untreated controls. The crystal violet assay was used to quantify the resulting biofilm biomass compared with untreated controls. An ELISA reader (Sunrise R4, Tecan) calculated the OD of the biofilm at 590 nm after the negative control (OD_NC_) was set at zero. The negative and positive controls used were sanitized medium only and the working solution, respectively (Pierce et al., 2008; Abou Elez et al., 2023). The rate (%) of biofilm inhibition was measured through the subsequent equation:


$$Biofilm\;inhibition\;rate\;\left(\%\right)\;=1-\;\frac{OD_{590}\;of\;treated\;cells\;}{OD_{590}\;of\;nontreated\;control\;}\times 100$$

#### 2.7.4 Antifungal effect

The antifungal efficacy of ZnNPs, probiotic *L. salivarius*, and ZnNCs was assessed against *C. albicans* isolates using the agar well diffusion method (CLSI, 2008). In brief, 25 mL of SDA agar was individually inoculated with 100 µL (0.5 × 10^^3^ CFU/mL) of *C. albicans* isolates. The mixture was then added into a Petri dish and allowed to settle at room temperature for 30 min. Subsequently, a 10 mm diameter well was created in the agar using a sterile corkborer. The wells were supplied with 100 µg of each ZnNPs, probiotic *L. salivarius*, and ZnNCs. Wells filled with (20 µg) and broth media served as positive and negative controls, respectively. The plates were adjusted for 48 h at 37°C, and the diameters of the inhibition zones were measured using a ruler.

The minimum inhibitory concentration (MIC) values of ZnNPs, probiotic *L. salivarius*, and ZnNCs against *C. albicans* were calculated by agar well diffusion technique as mentioned above. Different concentrations ranging from 5 to 100 µg/mL of each ZnNPs, probiotic *L. salivarius*, and ZnNCs were applied to the wells, and the plates were adjusted for 2 days at 37°C. The lowest agent concentration that inhibited germination parallels to the positive control after 48 h of incubation at 37°C was considered the MIC. The test was conducted in triplicate.

#### 2.7.5 Quantitative analysis of biofilm gene expression

Transcript expression was used to assess the effect of the tested factors on *C. albicans* adhesion (*ALS3*), filamentation (*HWP1*), the hyphal regulator (*RAS1*), and hyphal formation and virulence (*SAP4*), as previously described (Tsang et al., 2012). A 1 mL suspension of each *C. albicans* strain, OQ150021 and OQ150022, was transferred to the wells of pre-sterilized 24-well microtiter plates with a flat bottom and incubated at 37°C for 1.5 h under agitation. The wells were washed with PBS. Fresh RPMI 1640 medium (1 mL) containing MIC-50 of ZnNPs, probiotic *L. salivarius*, ZnNCs, and AMB was added to each well, and the plate was further incubated for 24 h at 37°C. The control wells were used for comparison. The wells were washed twice with PBS and 600 mL of RLT buffer was added to the wells. The plate was incubated for 10 min, and then transferred to 1.5 mL microcentrifuge tubes. For sample homogenization, a 2-min high-speed (30-Hz) tissue lyser was used.

For each harvested fungal culture, including untreated fungal culture (control) and the treated fungal culture (ZnNPs, probiotic *L. salivarius*, ZnNCs, and AMB-treated *C. albicans*), one volume was added to one volume of RNA protection reagent at each sampling time (Qiagen, Hilden, Germany) following the manufacturer’s instructions. RNA isolation was carried out using the QIAamp RNeasy Mini Kit (Qiagen, Hilden, Germany) following the manufacturer’s guidelines. SYBR Green I-based real-time PCR with specific primers for *ALS3*, *HWP1*, *RAS1*, and *SAP4* virulence genes was used as previously described (Tsang et al., 2012). The *ITS* gene was employed as a housekeeping gene (Tarini et al., 2010). The PCR reaction contained 12.5 µL of SYBR Green PCR Master Mix (QIAGEN), 0.25 µL of reverse transcriptase (200 U/µL), 0.5 µL of each primer (20 pmoL), 8.25 µL of nuclease-free water, and 3 µL of RNA template. The amplification curves and cycle threshold (Ct) values were determined by Stratagene MX3005P software. The ΔΔCt method was performed, according to Yuan et al. (2006).

#### 2.7.6 Scanning electron microscopy analysis

The impact of ZnNCs on the morphology and structural integrity of *C. albicans* biofilm was examined using scanning electron microscopy (SEM) (Jeol 2100, Tokyo, Japan), following established protocols (Ramage et al., 2002). Initially, 2 mL suspension of *C. albicans* OQ150021 cells at a concentration of 1 × 10^6^ cells/mL in RPMI 1640 was incubated at 37°C for 24 h. Subsequently, the resulting biofilm was formed on sterile plastic coverslips (Nalge Nunc International) placed in 24-well tissue culture plates (Costar, Corning Inc., USA). The biofilm was treated with ZnNCs, washed with PBS, and then fixed in a solution of 0.1 M cacodylate buffer and 2.5% (vol/vol) glutaraldehyde (pH 7.2) overnight. After fixation, the cells were subjected to a series of ethanol washes for dehydration and air-dried in a desiccator. SEM analysis was conducted on the gold-coated samples using a Baltec SDC 050 sputter coater.

### 2.8 Data analysis

All statistical tests and data visualization were performed with R software (version 4.2.0). A dendrogram was generated with the “*hclust*” function from the stats package. One-way analysis of variance compared the effects of AMB, ZnNPs, probiotic *L. salivarius*, and ZnNCs on *C. albicans* biofilm formation and biofilm gene expression. *P*-values <0.05 were considered statistically significant.

## 3 Results

### 3.1 *Candida* spp. isolation and identification

*Candida* spp. were detected in 38 (12.7%) of the specimens, comprising 18 (18%) from Nile tilapia, 7 (7%) from fish wash water, and 13 (13%) from human sellers (**Table 1**). Four *Candida* spp. were isolated as *C. krusei*, *C. glabrata*, *C. parapsilosis* and with *C. albicans* (42.1%) being the most frequently recovered. Notably, when focusing on hand swabs from human sellers, *C. albicans* accounted for a high percentage, specifically 42.9%.

**Table 1:** Occurrence of *Candida* spp. in Nile tilapia, fish wash water and fish sellers.

Sample type	No. of samples	No. *Candida* spp. positive samples (%)	No. (%) of samples positive to			
			*C. albicans*	*C. krusei*	*C. parapsilosis*	*C. glabrata*
Nile tilapia	100	18 (18)	8 (44.4)	3 (17.6)	2 (11.8)	5 (29.4)
Fish wash water	100	7 (7)	3 (42.9)	2 (28.6)	1(14.3)	1(14.3)
Fish sellers	100	13 (13)	5 (38.5)	3 (21.4)	1 (7.1)	4 (28.6)
Total	300	38 (12.7)	16 (42.1)	8 (21.1)	4 (10.5)	10 (26.3)

### 3.2 Molecular characterization of *C. albicans* isolates

Biochemically identified *C. albicans* were molecularly confirmed by detecting the *ITS1* gene. *C. albicans* virulence-associated genes, including *RAS1*, *HWP1*, *ALS3*, and *SAP4*, were identified (**Table 2**). The most frequently identified gene was *ALS3* (81.3%), followed by *HWP1* (56.3%), *RAS1* (50%), and *SAP4* (31.3%).

**Table 2:** Virulotypes, antifungal resistance patterns and biofilm degree of *C. albicans* isolates recovered from Nile tilapia, fish wash water and fish sellers.

ID	Source	Virulence genes^1^				Genotyping		Antifungal resistance^2^		Biofilm degree^3^
		*RAS1*	*HWP1*	*ALS3*	*SAP4*	Profile	Cluster	Patterns	MAR index	
F1	Tilapia	−	+	+	+	R5	III	NYS, ITR, MIC	0.37	M
F2	Tilapia	+	+	+	−	R10	IV	FLZ, CAS	0.25	S
F3	Tilapia	−	−	+	−	R9	Single	NYS, FLZ	0.25	M
F4	Tilapia	+	−	−	−	R7	Single	ITR, TER	0.25	M
F5	Tilapia	−	+	+	−	R2	I	FLZ, ITR, FUS, MIC	0.50	M
F6	Tilapia	+	+	+	−	R5	III	NYS, CAS	0.25	M
F7	Tilapia	+	−	−	−	R1	I	FLZ, CAS	0.25	M
F8	Tilapia	+	+	+	+	R10	IV	AMB, NYS, FLZ, FUS, CAS, MIC, TER	0.87	S
W9	Water	−	−	+	+	R2	I	NYS, ITR, TER	0.33	M
W10	Water	−	+	+	–	R2	I	NYS, ITR, MIC	0.33	M
W11	Water	+	–	–	+	R3	II	ITR, FUS	0.25	M
H12	Sellers	+	+	+	−	R3	II	AMB, FLZ	0.25	S
H13	Sellers	−	−	+	−	R4	II	NYS, FLZ, MIC	0.37	M
H14	Sellers	+	+	+	+	R8	Single	AMB, NYS, FLZ, ITR, FUS, CAS, TER	0.87	S
H15	Sellers	−	−	+	−	R6	Single	NYS, ITR, FUS	0.37	M
H16	Sellers	−	+	+	−	R1	I	AMB, NYS, CAS	0.37	M

^1^ +: virulence genes positive, −: virulence genes negative.

^2^ AMB, amphotericin B; NYS, nystatin; FLZ, fluconazole; ITR, itraconazole; FUS, flucytosine; CAS, caspofungin; MIC, micafungin; TER, terbinafine; MAR, multiple antibiotic index.

^3^ M, moderate biofilm forming; S, strong biofilm forming.

**Figure 1:**
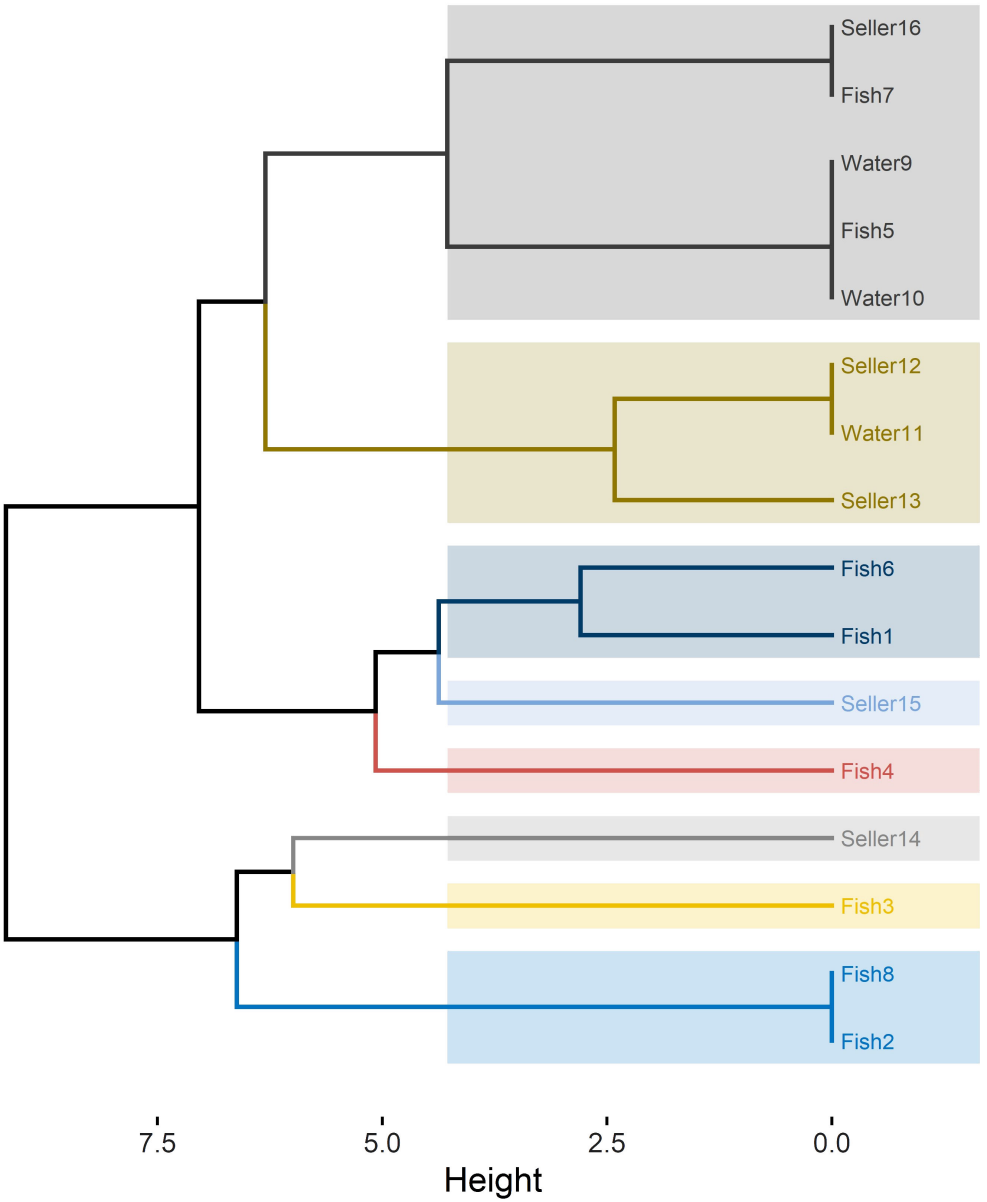
RAPD-PCR dendrogram for *C. albicans* isolates recovered from Nile tilapia, fish wash water, and fish sellers.

RAPD-PCR analysis of the 16 C*. albicans* isolates revealed 10 distinct profiles, denoted as R1 to R10 (**Table 2**). The power of RAPD-PCR was high, with a D value equal to 0.95. Cluster I included isolates obtained from tilapia, fish wash water, and human sellers (**Figure 1**). The similarity between the isolates from sellers and those from tilapia and water in clusters I and II was 100%, as determined by the Jaccard coefficient.

**Figure 2:**
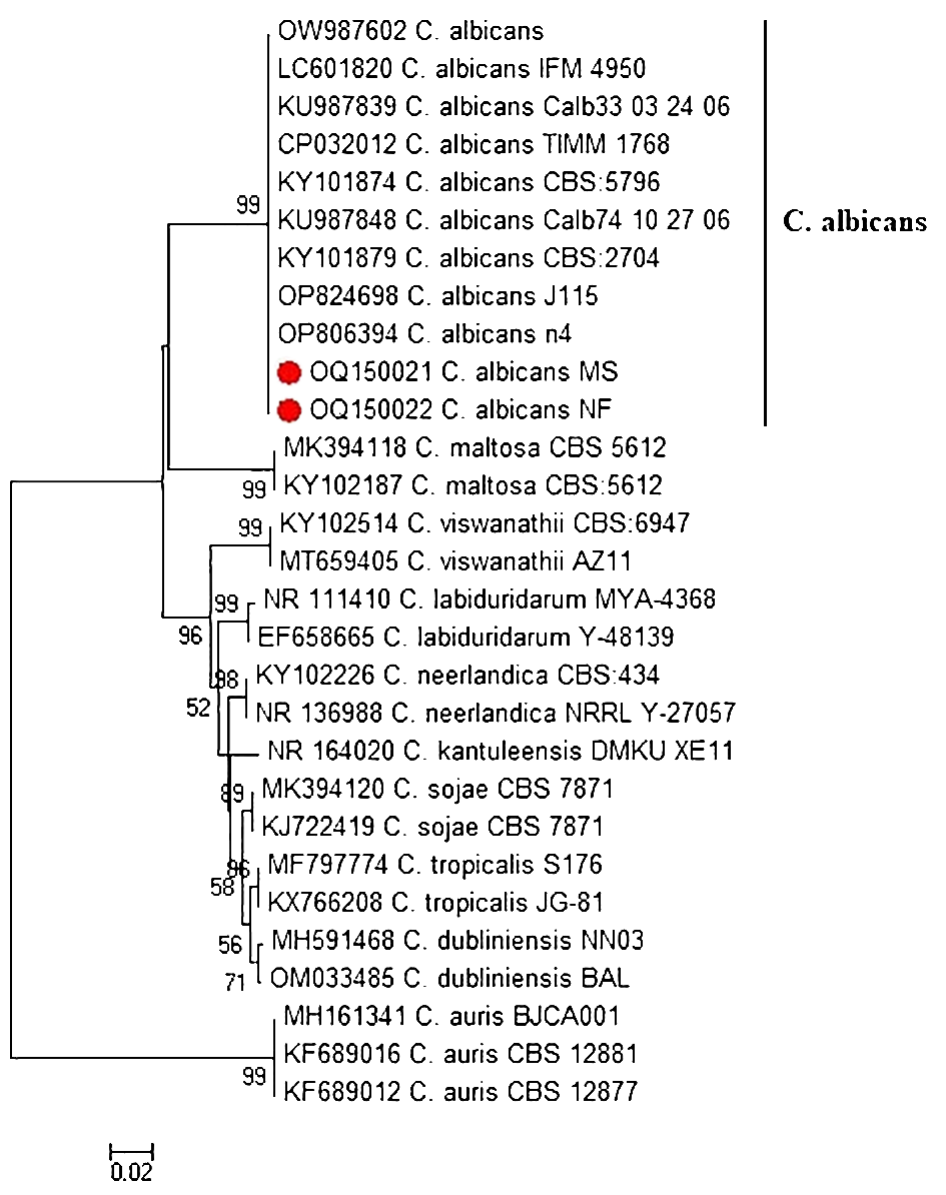
Phylogenetic tree of *C. albicans* isolated from Nile tilapia and fish seller (red dot) based on *ITS* gene sequences. A phylogenetic tree was generated using the neighbor-joining approach and 1000 bootstrap values.

The *ITS* region of two representative virulent biofilm-forming *C. albicans*, obtained from tilapia and human sellers, was sequenced and subsequently listed under accession numbers OQ150021 to OQ150022. Phylogenetic tree analysis revealed that the *ITS* sequences of *C. albicans* strains of fish and human sellers were associated with the lineage of other *C. albicans* from GenBank and were distinctly separated from other *Candida* spp. (**Figure 2**).

### 3.3 Antifungal susceptibility of *C. albicans*

The antifungal resistance patterns of *C. albicans* isolates to the 8 antifungal drugs are presented in **Tables 2** and **3**. Among the isolates, 62.5% exhibited resistance to at least one antifungal agent. Notably, the isolates demonstrated an increase in the degree of resistance to NYS (62.5%), followed by FLZ and ITR (50% each). Conversely, the isolates displayed a high level of susceptibility to AMB (75%). The MAR index ranged from 0.25 to 0.87, with an average of 0.56 (**Table 2**).

**Table 3:** Antifungal susceptibility results of 16 C*. albicans* isolates recovered from Nile tilapia, fish wash water, and fish sellers.

Antifungal class	Antifungals (µg/mL)^1^	Number (%) of *C. albicans*		
		Resistant	Intermediate	Susceptible
**Polyenes**	AMB (20 μg)	4 (25.0)	0 (0.0)	12 (75.0)
	NYS (100 U)	10 (62.5)	0 (0.0)	6 (37.5)
**Azoles**	FLZ (25 μg)	8 (50.0)	3 (18.8)	5 (31.2)
	ITR (10 μg)	8 (50.0)	6 (37.5)	2 (12.5)
**Flucytosine**	FUS (1 μg)	5(31.2)	4 (25.0)	7 (43.8)
**Echinocandins**	CAS (5 μg)	6 (37.5)	2 (12.5)	8 (50.0)
	MIC (1 µg)	5 (31.2)	3 (18.8)	8 (50.0)
**Allylamines**	TER (30 μg)	4 (25.0)	2 (12.5)	10 (62.5)

^1^ AMB, amphotericin B; NYS, nystatin; FLZ, fluconazole; ITR, itraconazole; FUS, flucytosine; CAS, caspofungin; MIC, micafungin; TER, terbinafine.

### 3.4 *C. albicans* biofilm formation ability

All *C. albicans* isolates exhibited biofilm-forming capabilities, with 4 (25%) isolates displaying strong biofilm formation (**Table 2**). However, the remaining 12 (75%) isolates showed moderate biofilm formation abilities.

### 3.5 Effects of probiotic *L. salivarius*, ZnNPs and ZnNCs on *C. albicans*

#### 3.5.1 Characterization of ZnNPs and ZnNCs

**Figure 3:**
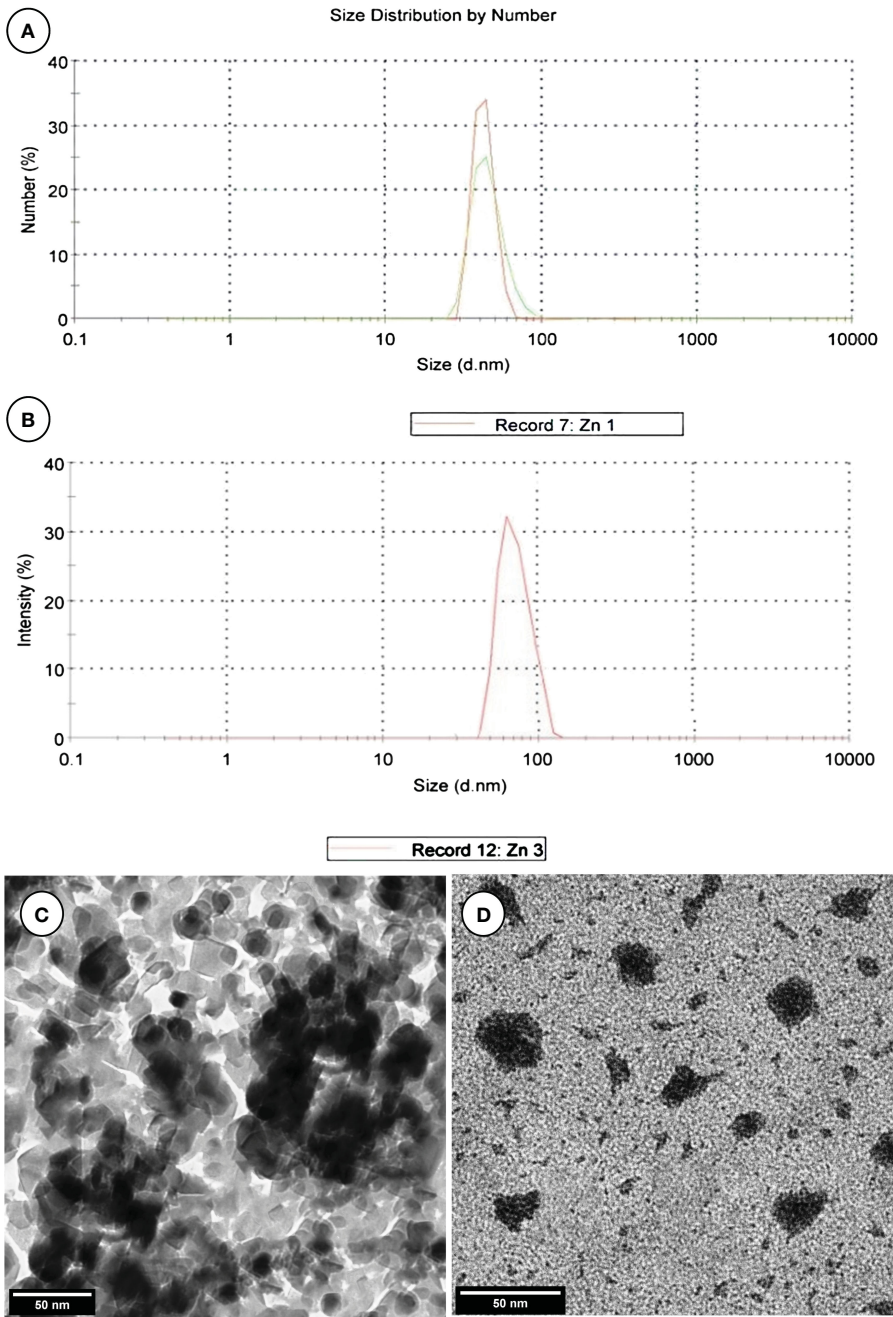
Characterization of ZnNPs and ZnNCs. DLS diameter of ZnNPs **(A)** and ZnNCs **(B)**; transmission electron microscopy of ZnNPs **(C)** and ZnNCs **(D)**.

**Figure 4:**
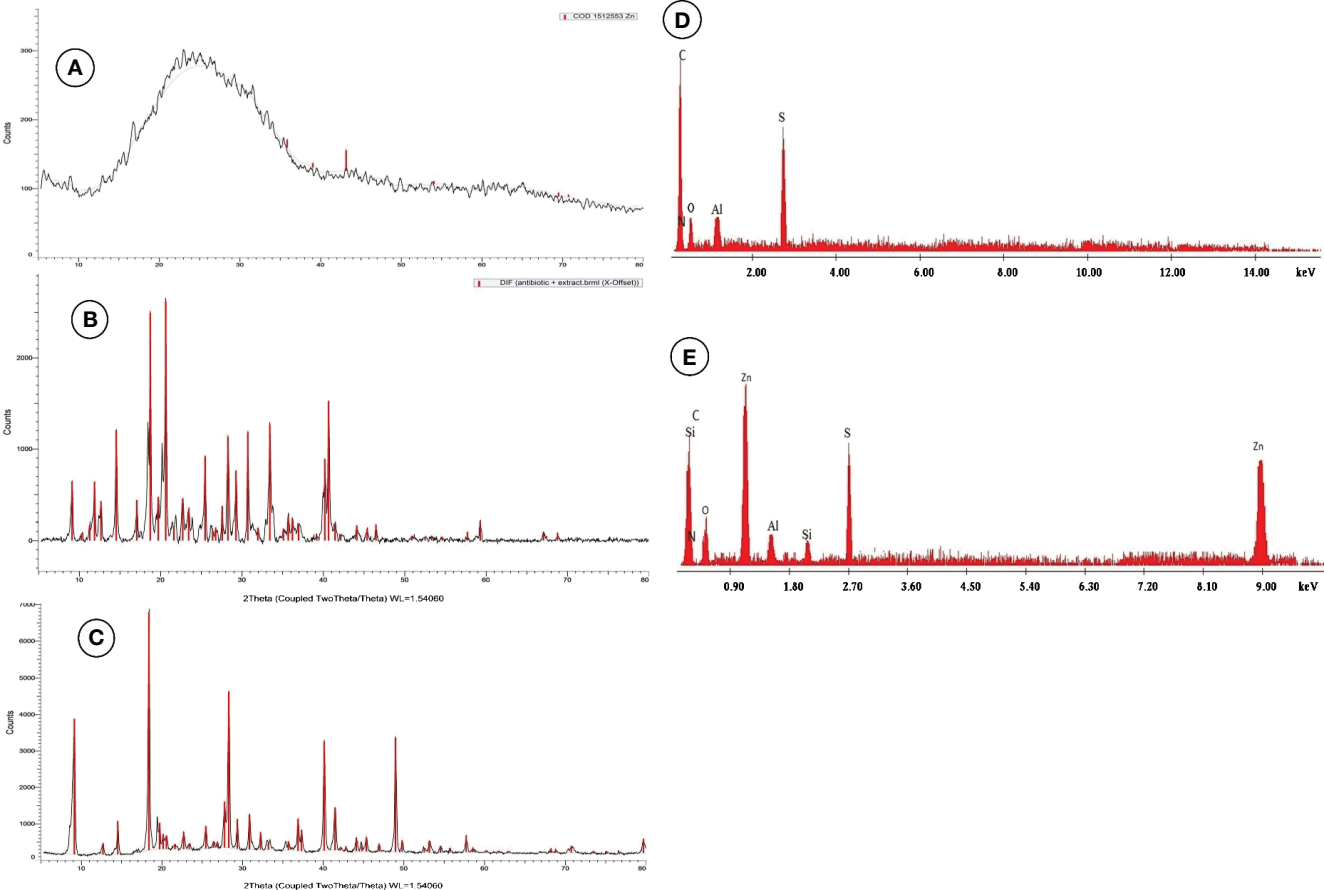
Powder X-ray diffraction (XRD) pattern for ZnNPs **(A)**, probiotic *L. salivarius* **(B)**, and ZnNCs **(C)**, and energy-dispersive X-ray spectroscopy (EDX) pattern for probiotic *L. salivarius* **(D)** and ZnNCs **(E)**.

ZnNPs were biosynthesized by *Aspergillus fumigatus* and have a characteristic peak at 45.82 nm (**Figure 3A**), however, ZnNCs had a characteristic peak at 85 nm (**Figure 3B**). Under TEM, ZnNPs exhibited spherical and cubic shapes (**Figure 3C**), while the ZnCNs have sub-rectangular shapes (**Figure 3D**). X-ray diffraction patterns for ZnNPs, probiotic *L. salivarius*, and ZnNCs are presented in **Figure 4**. ZnNPs displayed characteristic peaks at 2θ angles of 35.862°, 39.044°, 43.176°, 54.659°, 69.507°, and 76.012° (**Figure 4A**). Probiotic *L. salivarius* exhibited characteristic peaks at 2θ angles of 9.145°, 18.398°, 28.305°, 41.484°, 49.805°, 58.560°, 60.231°, 68.803°, and 79.697° (**Figure 4B**). On the other hand, ZnNCs displayed sharp characteristic peaks at 2θ angles of 9.124°, 14.558°, 18.734°, 20.637°, 28.284°, 33.442°, 40.194°, 40.662°, 54.659°, 59.291°, and 68.802°, indicating a cubic lattice of nanocomposites (**Figure 4C**). The elemental composition of the surface of the probiotic *L. salivarius* and ZnNCs was analyzed using EDS. In the spectrum of probiotic *L. salivarius*, carbon (55.10%), nitrogen (1.8%), oxygen (31.9%), calcium (9.4%), and silicon (1.79%) were found (**Figure 4D**). In the spectrum of ZnNCs, carbon (49.10%), nitrogen (1.75%), oxygen (27.79%), calcium (9.3%), silicon (1.89%), and zinc (9.35%) were identified (**Figure 4E**).

#### 3.5.2 Antibiofilm effect

**Figure 5:**
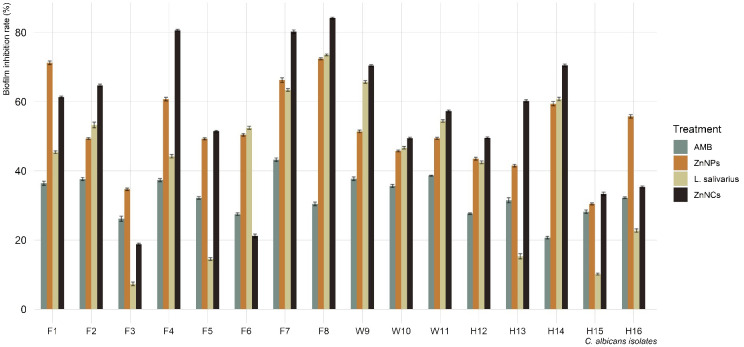
Biofilm inhibition rate of amphotericin B (AMB), ZnNPs, probiotic *L. salivarius* and ZnNCs against *C. albicans* isolates recovered from Nile tilapia, fish wash water, and fish sellers.

**Figure 5** displays the biofilm inhibition rates of AMB, ZnNPs, probiotic *L*. *salivarius*, and ZnNCs on *C. albicans* isolates recovered from tilapia, fish wash water, and fish sellers at 37°C. ZnNCs exhibited the highest biofilm inhibition rate at 84.4%, followed by ZnNPs. *C. albicans* treated with AMB displayed a significantly lower biofilm inhibition rate compared to those treated with ZnNPs, probiotic *L. salivarius*, and ZnNCs. Moreover, a significant difference in biofilm inhibition rate was observed when *C. albicans* was treated with probiotic *L. salivarius* and ZnNPs (*P* = 0.012) and ZnNCs (*P* = 0.0003). On the other hand, no significant variation was found within ZnNPs and ZnNCs (*P* = 0.685).

#### 3.5.3 Antifungal effect

The antifungal effects of ZnNPs, probiotic *L. salivarius*, and ZnNCs against *C. albicans* isolates recovered from tilapia and fish sellers were assessed using the disc diffusion method (**Table 4**). AMB, which was sensitive to most of the isolates (75%), was used as a positive control. ZnNPs, probiotic *L. salivarius*, and ZnNCs exhibited significantly larger inhibition zones than AMB (20 μg) for all *C. albicans* isolates. Furthermore, the MIC for inhibition of *C. albicans* growth was 10 µg/mL for ZnNCs and 20 µg/mL for both ZnNPs and probiotic *L. salivarius* (**Supplementary Table S2**).

**Table 4:** Antifungal activity of amphotericin B (AMB), ZnNPs, probiotic *L. salivarius* and ZnNCs against *C. albicans* isolates recovered from Nile tilapia, fish wash water, and fish sellers.

C. albicans		Inhibition zone diameter (mm) of 100 µg/mL concentration of			
Source	ID	AMB	ZnNPs	L. salivarius	ZnNCs
**Tilapia**	F1	19.1 ± 0.2	35.5 ± 0.5	36.3 ± 0.3	38.1 ± 0.7
	F2	21.3 ± 0.3	31.1 ± 0.8	38.8 ± 0.2	41.8 ± 0.5
	F3	21.2 ± 0.3	31.2 ± 1.0	40.3 ± 1.3	43.4 ± 0.2
	F4	18.2 ± 0.2	27.6 ± 0.4	39.0 ± 0.5	43.0 ± 0.6
	F5	19.2 ± 0.3	29.0 ± 0.5	40.2 ± 0.4	44.2 ± 0.2
	F6	19.3 ± 0.3	33.6 ± 0.8	40.7 ± 0.3	43.1 ± 0.5
	F7	18.8 ± 0.8	30.3 ± 0.7	41.8 ± 0.7	46.4 ± 0.4
	F8	18.3 ± 0.3	39.1 ± 0.2	44.2 ± 0.2	50.2 ± 0.2
**Wash water**	W9	18.1 ± 0.2	30.3 ± 1.8	38.0 ± 0.7	48.2 ± 0.2
	W10	20.1 ± 0.2	36.3 ± 0.3	37.7 ± 0.6	47.2 ± 0.2
	W11	20.3 ± 0.6	31.2 ± 0.3	38.3 ± 0.4	44.6 ± 0.5
**Sellers**	H12	20.2 ± 0.3	33.4 ± 0.4	39.4 ± 1.1	45.6 ± 0.5
	H13	20.3 ± 0.6	34.8 ± 0.3	38.3 ± 1.2	46.7 ± 0.6
	H14	18.0 ± 0.0	36.8 ± 0.1	42.4 ± 0.1	48.1 ± 0.2
	H15	18.3 ± 0.4	35.3 ± 0.3	39.4 ± 0.4	47.1 ± 0.3
	H16	21.2 ± 0.3	37.1 ± 0.3	34.6 ± 0.6	48.3 ± 0.3

#### 3.5.4 Effect on biofilm gene expression

**Figure 6:**
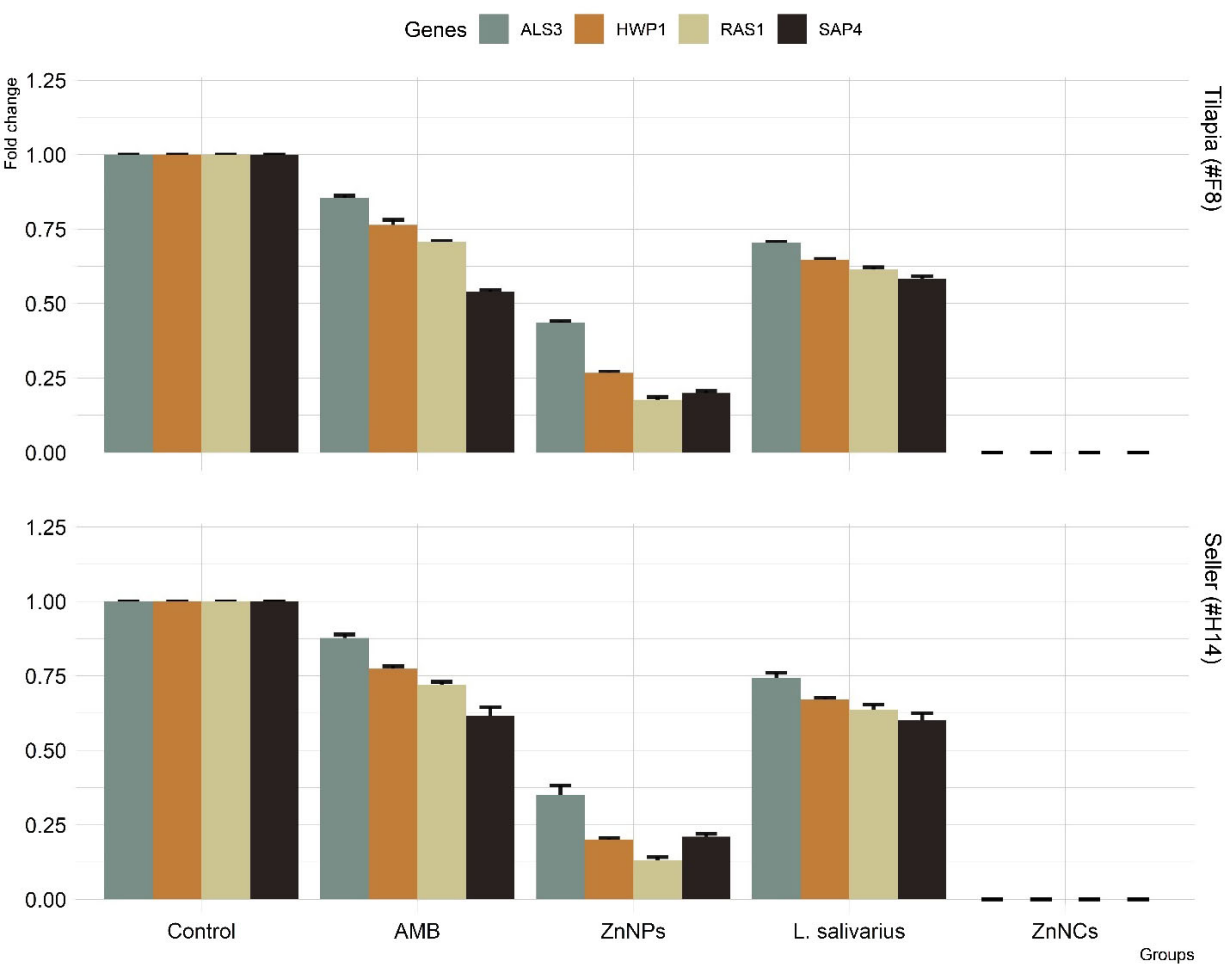
The relative mRNA expression levels of *C. albicans* adhesion (*ALS3*), filamentation (*HWP1*), hyphal regulator (*RAS1*), and hyphal formation and virulence (*SAP4*), before and after treatment with amphotericin B (AMB), ZnNPs, probiotic *L. salivarius* and ZnNCs.

**Figure 6** displays the particular gene expression patterns of four biofilm-forming genes (*ALS3*, *HWP1*, *RAS1*, and *SAP4*) in two *C. albicans* resistant isolates with strong biofilm-forming abilities that were subsequent tested with AMB, ZnNPs, probiotic *L. salivarius*, and ZnNCs. Treatment with ZnNPs significantly decreased the expression of the four biofilm genes compared to non-tested control isolates. However, ZnNCs showed a complete reduction in biofilm gene expression.

#### 3.5.5 SEM analysis

**Figure 7:**
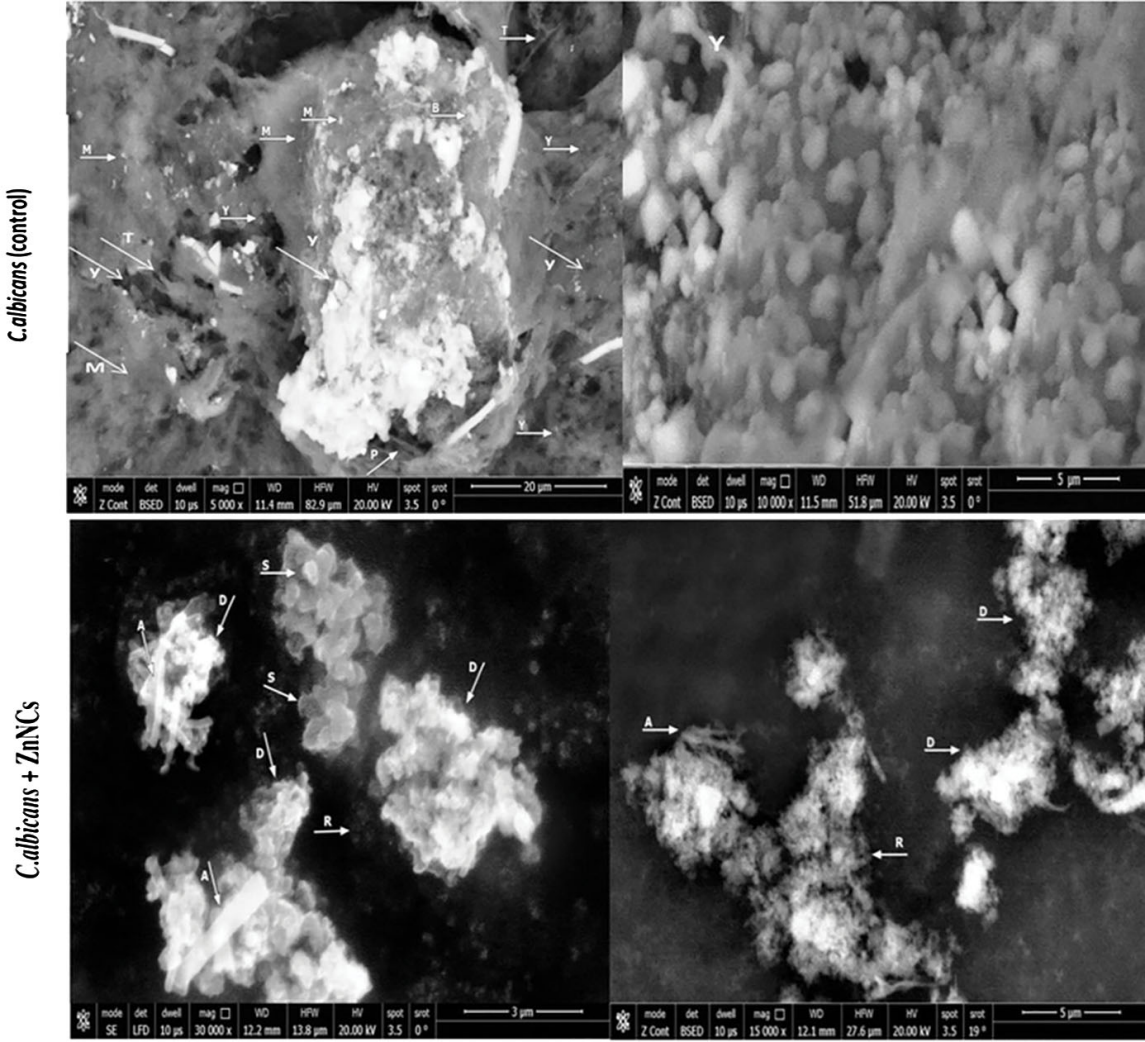
Scanning electron micrographs of biofilm formation by *C. albicans*. *C. albicans* (Control): showing mature biofilm with (M): multilayer of extracellular matrix, (Y): Yeast cells, (T): True hyphae, (B): Bud scars, (P): pseudohyphae. Yeast cells, hyphae and bud scars are seen encased, conglomerate and embedded in matrix material (M). Treated *C. albicans* with ZnNCs: showing (S): small shrinkage blastoconidia and the cell wall presented a rugged surface (A): absence of pseudohyphae, (D): Destruction, swollen and rupture of cell membranes of yeast cells, the outer layer seemed to detach from the cell and masses of cellular debris were seen with release of cell contents., (R): reduction of matrix layer.

SEM images of biofilm formation by *C. albicans* (control) and those treated with ZnNCs (**Figure 7**). Untreated cells displayed a typical mature biofilm with multiple layers of pseudohyphae, blastoconidia and an extracellular matrix forming a dense and heterogeneous polysaccharide network in which cells, pseudohyphae, and hyphae were embedded. Yeast germ tubes, hyphae, bud scars and pseudohyphae are encased in matrix material. The yeast layer and hyphal conglomerate development embedded in the matrix material are not visible and upper layer mainly the hyphal layer is indicated.

Conversely, *C. albicans* treated with 10 µg/mL ZnNCs exhibited the biofilm structure and prevented the formation of matrix material, resulting in an unusual biofilm architecture consisting of a single layer of loosely arranged yeast cells with noticeable morphological alterations, including small shrinkage of blastoconidia, absence of pseudohyphae, and reduction of the matrix layer.

## 4 Discussion

*Candida* spp. are commensal yeasts found on the gastrointestinal tract, skin, and other mucosal surfaces in healthy humans. These opportunistic pathogens are generally harmless to healthy individuals but can potentially lead to invasive diseases in immunocompromised individuals (Pappas et al., 2018). *C. albicans* is typically present in the human microbiota and is commonly associated with organic matter. However, this opportunistic disease is known to increase the prevalence of pathogenicity in immunocompromised persons (Asadzadeh et al., 2022). In our study, *Candida* spp. were identified in 12.7% of the samples, with 18% originating from tilapia, which aligns with the finding of a previous Egyptian study in which *Candida* spp. were isolated from tilapia, catfish, and gray mullet (Tartor et al., 2018). Among the identified *Candida* spp., *C. albicans* was the prevalent strain, consistent with findings in previous studies (Delgado et al., 2009; Hise et al., 2009; Junqueira et al., 2012; Li et al., 2013; Terças et al., 2017). The high isolation rate of *C. albicans* can be attributed to its remarkable adaptability, as *C. albicans* can thrive in vastly different environments characterized by varying nutrient availability, temperature fluctuations, pH levels, osmolarity, and oxygen concentrations (Paramythiotou et al., 2014).

In the current study, at least one of the virulence-associated genes (*ALS3*, *HWP1*, *RAS1*, and *SAP4*) was identified in all *C. albicans* isolates. Previous studies in Egypt have reported a higher prevalence of *HWP1* and *ALS1* genes in *C. albicans* isolates from patients (Shrief et al., 2019; Hamady and Marei, 2021). Additionally, another study revealed that *RAS1* and *ALS1* were present in all *Candida* isolates, while *HWP1* and *SAP4* were found in half of the isolates (Soliman et al., 2020). In addition, Inci et al. (2013) reported that 53.9% of recognized *C. albicans* strains had the *ALS1* gene and only 5.3% of recognized strains had the *HWP1* gene. The variation in the frequency of virulence genes among these studies may be attributed to differences in sample size, source of *C. albicans* isolates, the host, geographic location, the number of isolates studied, the site from which *C. albicans* was isolated, sample types, and diagnostic technique used (Vijayalakshmi et al., 2016).

Antifungal susceptibility testing is a crucial tool for determining clinical responses, facilitating the selection of appropriate antifungal agents, predicting treatment outcomes and studying the epidemiology of drug-resistant *C. albicans*. Monitoring the emergence of resistant isolates is essential for providing valuable information to clinicians for effective therapeutic decisions (Khan et al., 2018). In our current study, AMB is the preferred drug for treating candidiasis in animals, poultry, and humans in Egypt. However, most of the isolates in our study exhibited resistance to NYS, FLZ, and ITR, which consistent with the findings of previous studies (Costa-de-Oliveira and Rodrigues, 2020; Dhasarathan et al., 2021). In contrast, Sonmez and Erbas (2017) reported an increase in resistance (100%) to FLZ, AMB, and FUS, with high susceptibility to ketoconazole. Lyon et al. (2010) observed a significant increase in FLZ susceptibility in 2005 from 87.5% to 97.4% in 2007. Monroy-Pérez et al. (2016) found that all *C. albicans* isolates were susceptible to NYS, while 94.9% and 97.4% were resistant to FLZ and ketoconazole, respectively. Shrief et al. (2019) noted that resistance to antifungal agents, ITR, FLZ, and CAS, was 8% for each and 9% for AMB. Furthermore, Dos Santos Abrantes et al. (2014) reported that more than 50% of *C. albicans* isolates from South Africa and Cameroon exhibited resistance to FLZ. On the other hand, an earlier South African investigation revealed 100% susceptibility of *C. albicans* to FLZ.

Biofilm formation is essential for long-range colonization in host tissues and resistance to external stressors, such as oxidative stress and antifungal factors (Douglas, 2003; Sherry et al., 2017). Once a biofilm forms, candidal cells are encased in an extracellular matrix that confers resistance to fungicides and various sanitizing techniques (Ramage et al., 2009; Sherry et al., 2017). Innovative antibiofilm therapies are urgently needed to address these challenging conditions.

In our study, all *C. albicans* isolates demonstrated biofilm-forming abilities, consistent with the presence of biofilm-associated genes. Biofilm associated genes such as *ALS3*, *ALS1*, and *HWP1* within the *ALS* family are integral to the adhesion and biofilm formation processes of *Candida* spp. (Khan et al., 2010). These genes encode proteins that are essential for cell wall integrity and play a role in the initiation of hyphal development. *ALS3* and *HWP1* are cell wall proteins that play roles in intercellular adherence and cell-substrate interactions for proper formation of three-dimensional biofilm architectures (Nobile et al., 2006; Nobile et al., 2008). Previous studies in Egypt have reported that 84.6% of *C. albicans* isolates are capable of forming biofilms (Ismail et al., 2020a), while 58% of *C. albicans* isolated from intensive care unit patients with nosocomial infections exhibit biofilm-forming abilities (Shrief et al., 2019). The ability of *C. albicans* to form biofilms is a major part of most *C. albicans* infections (Nett and Andes, 2006), necessitating the development of alternative treatment strategies to combat biofilm-associated candidiasis.

Recently, there has been an interest in the use of nanoparticles and probiotics to counteract microbial pathogenicity, particularly related to biofilm formation (Lara et al., 2015; Hosseini et al., 2019; Sitohy et al., 2021). Various studies have shown the broad-spectrum actions of different probiotics, which can inhibit *C. albicans* proliferation, adhesion, and biofilm formation, making them valuable antimicrobial agents (Matsubara et al., 2016; Osman and El-Gazzar, 2021). *L. salivarius* exhibits an antifungal effect against *Candida* spp. through the production of lactic acid and organic acids that positively influence the efficacy of antifungal agents by increasing the permeability of the fungal plasma membrane structure followed by changes in gene expression and the transition to hyphal growth (Krzyściak et al., 2017; Zangl et al., 2019). In addition, *L. salivarius* showed a decrease in both the number of colonies and the biofilm biomass, as did the cross-linking of the biofilm structure (Zangl et al., 2019). Therefore, the development of safe antimicrobial probiotics with nanoparticle drug delivery capabilities is essential. Where, we investigated the inhibitory roles of ZnNPs, probiotic *L. salivarius*, and ZnNCs against *C. albicans* biofilm formation.

Zn-NPs have a long history of use in treating various ailments and have garnered significant interest from the scientific community (Hosseini et al., 2019). These agents have already demonstrated their potency as antiseptic and antimicrobial agents, making them particularly attractive for developing a new class of antimicrobial agents based on nanomaterials (Hosseini et al., 2019).

The DLS data showed a single peak at 45.82 nm for ZnNPs. TEM revealed an even distribution without agglomeration and a variety of shapes of ZnNPs in solution. This information may help explain the diversity of molecules involved in ZnNPs production, as these compounds can act as capping and agglomeration-preventing agents (El-Gazzar et al., 2021). X-ray analysis confirmed the appearance of a distinctive cubic lattice and peaks corresponding to the ZnNPs in a novel probiotic nanocomposite. The nanocomposite of probiotics with ZnNPs was further investigated by EDS surface area analysis, which supported previous findings (Sitohy et al., 2021).

The inhibitory effects of nanoparticles can vary depending on their size and concentration (Sitohy et al., 2021). In the present study, ZnNCs demonstrated significantly higher antifungal activity than ZnNPs, probiotics, or AMB. This finding aligns with Mare et al. (2021), who demonstrated a synergistic interaction when two agents are combined, resulting in an inhibitory effect greater than the sum of their individual effects. Another previous study revealed that the combination of FLZ or voriconazole with silver nanoparticles effectively treated drug-resistant *C. albicans*. The relationship between nanoparticle concentration and fungicidal action depends on the type of fungus (Li et al., 2018).

Biofilms are microbial colonization of tightly adherent cells to a surface that is embedded in a polymeric extracellular matrix. ZnNCs disrupted the ultrastructure of *C. albicans* biofilms due to their strong antifungal activity. Prior research has shown that nanoparticles alter fungal cell walls and membranes (Bonilla et al., 2017; Lee and Jun, 2019). In this study, ZnNCs prevented *C. albicans* from budding and forming germ tubes, similar to findings with silver nanocomposites (Guisbiers et al., 2017).

ZnNPs easily bind to biofilms and improve probiotic penetration, disrupting the lipidome. The antibiofilm activity of ZnNPs included inhibiting blastospores and hyphae and preventing *Candida* biofilm formation (Lara et al., 2015; Różalska et al., 2018). The inhibitory efficacy of probiotics is attributed to bacteriocins and organic acids. Bacteriocins’ positive charged sediment of amino acids creates pores and electrostatic difference in cell membranes, combined with cell lysis. Organic acids in probiotics reduce pH, creating an acidic environment where pathogenic *Candida* spp. cannot thrive (Osman and El-Gazzar, 2021).

*C. albicans*’ virulence depends on the ability of *C. albicans* to transition from yeast to hyphae, promoting adherence and biofilm formation. Inhibiting this transition can prevent infection (Modrzewska and Kurnatowski, 2015; Saibabu et al., 2020). A gene expression experiment showed that ZnNCs significantly decreased the expression of *C. albicans* genes, which are implicated in the yeast-to-hypha transition. This downregulation was observed in previous studies, and was associated with delayed or halted infection (Maubon et al., 2014; AJenull et al., 2017). In addition, previous studies have shown that the saturation of adhesion sites and coaggregation of *Lactobacillus* spp. prevent adherence of *C. albicans*. Gene expression in *C. albicans* changes due to the presence of *Lactobacillus*. The expression of genes responsible for adherence and yeast hyphal formation is reduced. The presence of *Lactobacillus* can alter the host immune response during *Candida* colonization to attract granulocytes and promote immune defense (Zangl et al., 2019). Thus, ZnNCs have the potential to target *C. albicans*’ virulence factors, suggesting that ZnNCs could be a novel therapeutic approach for treating candidiasis.

## 5 Conclusion

This study isolated *Candida* spp. from Nile tilapia, fish wash water, and human fish sellers in retail markets in Sharika Government, Egypt. *C. albicans* was the most frequently isolated *Candida* spp., with all isolates having at least one virulence gene, resistance to one antifungal agent, and moderate to strong biofilm-forming abilities, thus posing a significant risk to food safety and public health. The findings demonstrated that ZnNPs, probiotic *L. salivarius*, and ZnNCs have potential antifungal and antibiofilm activities against *C. albicans*. Our results suggested that ZnNCs could be promising, cost-effective antifungal drugs for inhibiting key virulence factors and preventing pathogenesis in *C. albicans*. In addition, ZnNCs demonstrated significantly higher antifungal activity than ZnNPs, probiotics, or AMB. However, further research is essential to determine the full potential of ZnNCs as robust antifungal and antibiofilm agents for managing critical *C. albicans* infections.

## Data availability statement

The original contributions presented in the study are included in the article/**Supplementary Material**. Further inquiries can be directed to the corresponding authors.

## Ethics statement

The studies involving humans were approved by Institutional Review Board under the number ZU-IRB #11274 -29/11-2023. The studies were conducted in accordance with the local legislation and institutional requirements. Written informed consent for participation in this study was provided by the participants’ legal guardians/next of kin. The animal study was approved by Institutional Animal Care and Use Committee (IACUC) of Zagazig University (Ref. No.: IACUC/2/F/234/2023). The study was conducted in accordance with the local legislation and institutional requirements.

## Author contributions

NE: Conceptualization, Data curation, Investigation, Methodology, Validation, Visualization, Writing – original draft, Writing – review & editing. RA: Conceptualization, Data curation, Investigation, Methodology, Resources, Validation, Writing – original draft, Writing – review & editing. AA: Conceptualization, Investigation, Methodology, Resources, Writing – review & editing. AA: Data curation, Funding acquisition, Resources, Validation, Writing – review & editing. MD: Methodology, Data curation, Investigation, Resources, Software, Validation, Visualization, Writing – review & editing. EY: Funding acquisition, Resources, Validation, Writing – review & editing. RE: Data curation, Investigation, Methodology, Resources, Validation, Visualization, Writing – review & editing. KM: Investigation, Methodology, Resources, Validation, Writing – review & editing. SD: Funding acquisition, Resources, Validation, Writing – review & editing. IE: Data curation, Formal analysis, Software, Validation, Visualization, Writing – original draft, Writing – review & editing.
