# Supplementary material for: Antifungal and antibiofilm effects of probiotic Lactobacillus salivarius, zinc nanoparticles, and zinc nanocomposites against Candida albicans from Nile tilapia (Oreochromis niloticus), water and humans
Source: Front Cell Infect Microbiol. 2024 Jun 4;14:1358270. doi: 10.3389/fcimb.2024.1358270 (PMC11183309; doi:10.3389/fcimb.2024.1358270)
Supplement: Supplementary file 1 [file Table_1.docx]

Supplementary Material

Antifungal and antibiofilm effect of probiotic *Lactobacillus salivarius*, zinc nanoparticles, and zinc nanocomposite against *Candida albicans* from Nile tilapia (*Oreochromis niloticus*), water and humans

**Nashwa El-Gazzar^1†^, Rasha M. M. Abou Elez^2†*^, Amira S. A. Attia^3^, Abdel-Wahab A. Abdel-Warith^4^, Manal M. Darwish^5,6^, Elsayed M. Younis^4^, Rehab A. Eltahlawi^7^, Kawthar Ibraheem Mohamed^5^, Simon J. Davies^8^, Ibrahim Elsohaby^9,10,11†*^**

^1^ Department of Botany and Microbiology, Faculty of Science, Zagazig University, Zagazig, Egypt

^2^ Department of Zoonoses, Faculty of Veterinary Medicine, Zagazig University, Zagazig, Egypt

^3^ Department of Veterinary Public Health, Faculty of Veterinary Medicine, Zagazig University, Zagazig, Egypt

^4^ Department of Zoology, College of Science, King Saud University, Riyadh, Saudi Arabia

^5^ Medical Microbiology Department, Faculty of Medicine, Ain Shams University, Cairo, Egypt,

^6^ Microbiology and Immunology Department, Faculty of Pharmacy, October University for Modern Sciences and Arts, Giza, Egypt,

^7^ Microbiology and Immunology Department, Faculty of Medicine, Zagazig University, Zagazig, Egypt

^8^ Aquaculture Nutrition Research Unit ANRU, Carna Research Station, Ryan Institute, College of Science and Engineering, University of Galway, Galway, Ireland

^9^ Department of Infectious Diseases and Public Health, Jockey Club College of Veterinary Medicine and Life Sciences, City University of Hong Kong, Hong Kong, Hong Kong SAR, China

^10^ Centre for Applied One Health Research and Policy Advice (OHRP), City University of Hong Kong, Hong Kong, Hong Kong SAR, China

^11^ Department of Animal Medicine, Faculty of Veterinary Medicine, Zagazig University, Zagazig, Egypt

^†^ These authors contributed equally to this work

*** Correspondence:**Ibrahim Elsohaby ([ielsohab@cityu.edu.hk](mailto:ielsohab@cityu.edu.hk)) and Rasha M. Abou Elez ([rmmohamed@zu.edu.eg](mailto:rmmohamed@zu.edu.eg))

# Supplementary Figures and Tables

## Supplementary Table 1S: Nucleotide sequences and product sizes of *Candida* genes primers.

| **Target gene** | **Primers sequences 5`-3`** | **Amplified segment (bp)** | **References** |
| --- | --- | --- | --- |
|  |  |  |  |
| *ITS1* | GGTTTGCTTGAAAGACGGTAG | 109 | Tarini et al. (2010) |
|  | AGTTTGAAGATATACGTGGTAG |  |  |
| *ALS3* | CTGGACCACCAGGAAACACT | 122 | Tsang et al. (2012) |
|  | ACCTGGAGGAGCAGTGAAAG |  |  |
| *HWP1* | ATG ACT CCA GCT GGT TC | 572 | İnci et al. (2013) |
|  | TAG ATC AAG AAT GCA GC |  |  |
| *RAS1* | CCCAACTATTGAGGATTCTTATCGTAAA | 106 | Tsang et al. (2012) |
|  | TCTCATGGCCAGATATTCTTCTTG |  |  |
| *SAP4* | GCT CTT GCT ATT GCT TTA TTA | 394 | Sikora et al. (2011) |
|  | TAG GAA CCG TTA TTC TTA CA |  |  |
| *OPA-18* | AGCTGACCGT | -- | Bautista-Muñoz et al. (2003) |
| *ITS* | ITS1: TCCGTAGGTGAACCTGCGG | variable | Tarini et al. (2010) |
|  | ITS4: TCCTCCGCTTATTGATATGC |  |  |

**References:**

Bautista-Muñoz, C., Boldo, X. M., Villa-Tanaca, L. , and Hernández-Rodríguez, C. (2003). Identification of *Candida* spp. by randomly amplified polymorphic DNA analysis and differentiation between *Candida albicans* and *Candida dubliniensis* by direct PCR methods. *J. Clin. Microbiol.* 41**,** 414-420. <https://doi.org/10.1128/JCM.41.1.414-420.2003>.

Inci, M., Atalay, M. A., Özer, B., Evirgen, Ö., Duran, N., Motor, V. K., et al. (2013). Investigations of *ALS1* and *HWP1* genes in clinical isolates of *Candida albicans*. *Turk. J. Med. Sci.* 43**,** 125-130.

Sikora, M., Dabkowska, M., Swoboda-Kopec, E, Jarzynka, S., Netsvyetayeva, I., Jaworska-Zaremba, M., et al. (2011). Differences in proteolytic activity and gene profiles of fungal strains isolated from the total parenteral nutrition patients. Folia *Microbiol. (Praha)* 56**,** 143-148. <https://doi.org/10.1007/s12223-011-0023-3>.

Tarini, N., Wahid, M., Ibrahim, F., Yasmon, A., and Djauzi, S. (2010). Development of multiplex-PCR assay for rapid detection of *Candida* spp. *Med. J. Indones* 19 (2)**,** 83-87. <https://doi.org/10.13181/mji.v19i2.387>.

Tsang, P. W., Bandara, H. M., and Fong, W. P. (2012). Purpurin suppresses *Candida albicans* biofilm formation and hyphal development. *PLoS One* 7**,** e50866. <https://doi.org/10.1371/journal.pone.0050866>.

## Supplementary Table 2S: Minimum inhibitory concentration (MIC) of ZnNPs, probiotic *L. salivarius* and ZnNCs against two *C. albicans* isolates recovered from tilapia and fish sellers

| **Treatment** | **Concentration (μg/mL)** | **Inhibition zone diameter (mm) of *C.* *albicans* isolates** | |
| --- | --- | --- | --- |
|  |  | **H14 (OQ150021)** | **F8 (OQ150022)** |
| **ZnNPs** | 5 | 0 | 0.0 |
|  | 10 | 0 | 0.0 |
|  | 20 | 18.1 ± 0.2 | 18.2 ± 0.2 |
|  | 30 | 23.2 ± 0.2 | 22.3 ± 0.1 |
|  | 40 | 29.2 ± 0.2 | 30.3 ± 0.1 |
|  | 50 | 33.1 ± 0.2 | 34.3 ± 0.1 |
|  | 70 | 35.4 ± 0.1 | 37.1 ± 0.2 |
|  | 100 | 36.8 ± 0.1 | 39.3 ± 0.3 |
| ***L. salivarius*** | 5 | 0 | 0.0 |
|  | 10 | 0 | 0.0 |
|  | 20 | 18.2 ± 0.2 | 18.3 ± 0.2 |
|  | 30 | 25.1 ± 0.2 | 31.3 ± 0.1 |
|  | 40 | 33.2 ± 0.2 | 37.4 ± 0.3 |
|  | 50 | 36.2 ± 0.2 | 41.4 ± 0.2 |
|  | 70 | 38.4 ± 0.1 | 43.1 ± 0.2 |
|  | 100 | 42.4 ± 0.1 | 44.5 ± 0.4 |
| **ZnNCs** | 5 | 0.0 | 0.0 |
|  | 10 | 26.7 ± 0.1 | 31.2 ± 0.2 |
|  | 20 | 33.1 ± 0.2 | 36.3 ± 0.3 |
|  | 30 | 39.2 ± 0.2 | 41.2 ± 0.7 |
|  | 40 | 44.1 ± 0.1 | 46.3 ± 0.3 |
|  | 50 | 46.9 ± 0.3 | 49.5 ± 0.4 |
|  | 70 | 47.5 ± 0.5 | 50.1 ± 0.2 |
|  | 100 | 48.7 ± 0.4 | 54.2 ± 0.3 |
